# Lysosomes Dysfunction Causes Mitophagy Impairment in PBMCs of Sporadic ALS Patients

**DOI:** 10.3390/cells11081272

**Published:** 2022-04-09

**Authors:** Matteo Bordoni, Orietta Pansarasa, Eveljn Scarian, Riccardo Cristofani, Roberta Leone, Valentina Fantini, Maria Garofalo, Luca Diamanti, Stefano Bernuzzi, Stella Gagliardi, Stephana Carelli, Angelo Poletti, Cristina Cereda

**Affiliations:** 1 Genomic and Post-Genomic Unit, IRCCS Mondino Foundation, 27100 Pavia, Italy; matteo.bordoni@mondino.it (M.B.); eveljn.scarian@mondino.it (E.S.); maria.garofalo@mondino.it (M.G.); stella.gagliardi@mondino.it (S.G.); cristina.cereda@asst-fbf-sacco.it (C.C.); 2 Department of Brain and Behavioral Sciences, University of Pavia, 27100 Pavia, Italy; 3 Dipartimento di Scienze Farmacologiche e Biomolecolari (DiSFeB), Dipartimento di Eccellenza 2018-2022, Università Degli Studi di Milano, 20133 Milano, Italy; riccardo.cristofani@unimi.it (R.C.); angelo.poletti@unimi.it (A.P.); 4 Casa di Cura Ars Medica S.p.a, 00191 Rome, Italy; roberta.leone02@universitadipavia.it; 5 Laboratory of Neurobiology and Neurogenetic, Golgi-Cenci Foundation, 20081 Abbiategrasso, Italy; v.fantini@golgicenci.it; 6 Neuroncology Unit, IRCCS Mondino Foundation, 27100 Pavia, Italy; luca.diamanti@mondino.it; 7 Immunohematological and Transfusional Service and Centre of Transplantation Immunology, IRCCS “San Matteo Foundation”, 27100 Pavia, Italy; s.bernuzzi@smatteo.pv.it; 8 Department of Biomedical and Clinical Sciences “L. Sacco”, University of Milan, 20157 Milan, Italy; stephana.carelli@unimi.it; 9 Pediatric Clinical Research Centre Fondazione “Romeo ed Enrica Invernizzi”, University of Milano, 20157 Milan, Italy

**Keywords:** ALS, PBMCs, mitochondria, trehalose, mTOR, autolysosomes

## Abstract

Mitochondria alterations are present in tissues derived from patients and animal models, but no data are available for peripheral blood mononuclear cells (PBMCs) of ALS patients. This work aims to investigate mitophagy in PBMCs of sporadic (sALS) patients and how this pathway can be tuned by using small molecules. We found the presence of morphologically atypical mitochondria by TEM and morphological abnormalities by MitoTracker™. We found a decreased number of healthy mitochondria in sALS PBMCs and an impairment of mitophagy with western blot and immunofluorescence. After rapamycin treatment, we found a higher increase in the LC3 marker in sALS PBMCs, while after NH4Cl treatment, we found a lower increase in the LC3 marker. Finally, mTOR-independent autophagy induction with trehalose resulted in a significant decrease in the lysosomes level sALS PBMCs. Our data suggest that the presence of morphologically altered mitochondria and an inefficient turnover of damaged mitochondria in PBMCs of sALS patients rely on the impairment of the mitophagy pathway. We also found that the induction of the mTOR-independent autophagy pathway leads to a decrease in lysosomes level, suggesting a more sensitivity of sALS PBMCs to trehalose. Such evidence suggests that trehalose could represent an effective treatment for ALS patients.

## 1. Introduction

Amyotrophic lateral sclerosis (ALS) is a rare adult-onset motor neuron disorder characterized by a fatal progressive degeneration of the upper and lower motor neurons at the spinal and bulbar spinal cord level [1]. Motor neuron degeneration results in progressive muscle weakness and death, usually occurring by respiratory failure on average 2–3 years post-onset. The median incidence rate of ALS is approximately 2–3/100,000 in Europe, with a mean age of onset between 50 and 65 years [1]. The majority of the cases (90–95%) are classified as sporadic forms of ALS (sALS), which have no evident genetically inherited component, while the remaining 5–10% of the cases are familial forms of ALS (fALS) [2]. More than 20 genes have been associated with the disease, including superoxide dismutase 1 (SOD1), TAR DNA binding protein (TARDBP), fused in sarcoma/translocated in liposarcoma (FUS/TLS), and C9ORF72 repeat expansions. These mutations can be found not only in fALS but also in sALS [3,4]. Genes involved in ALS pathogenesis belong to a wide range of cellular mechanisms suggesting that ALS is a multifactorial disease [5,6].

Mitochondrial dysfunction is considered an important mechanism in the pathogenesis of neurodegenerative diseases, including ALS; in fact, mitochondria play a central role in cell survival and metabolism, being the organelle responsible for ATP production, calcium buffering, and apoptotic signaling [7]. Mitochondria are essential in neurons because of the high demand of energy of the brain, consuming 20% of total ATP produced by the organism despite this organ representing only 2% of the body mass [8]. Functional defects and altered mitochondria morphology such as fragmented network, swelling, and augmented cristae were found in the soma and proximal axons of skeletal muscle and spinal motor neurons of ALS patients [9,10]. Moreover, mislocalization and aggregation of mitochondria have been observed in several districts of motor neurons of ALS patients (i.e., the soma, dendrites, and proximal axons), suggesting that the regulation of mitochondria transport along axons is crucial for neuron survival. Taken together, these observations suggest that the dysfunction of mitochondria transport is a recurrent ALS feature [5,11].

Mitochondria are known to be highly dynamic organelles that form a network undergoing fusion and fission events in response to various stimuli and cues [12]. Generally, in healthy conditions, the mitochondrial network is regulated by the interplay between Dynamin-related protein 1 (DRP1) and Fission 1 (FIS1) that promote fission, while Mitofusin 1 and 2 (MFN1, MFN2), and Optic atrophy 1 (OPA1) promote fusion [13]. The role of the mitochondrial network in ALS pathogenesis is still unclear as some evidence suggests that inhibition of fission increases motor neuron viability [14], while other groups reported that neuronal survival promotes mitochondrial fusion [15]. However, it is now clear that the imbalance of fission/fusion processes leads to mitochondrial fragmentation of mitochondria.

Furthermore, a selective form of macroautophagy, called mitophagy, physiologically removes damaged mitochondria. Loss of mitochondrial membrane potential (MMP) and damaging of mitochondria leads to accumulation of PTEN-induced kinase 1 (PINK1) in the outer membrane (OM), where it recruits Parkin that activates the removal of mitochondria by autophagosomes [16]. In addition, recent studies have identified critical roles for some ALS genes. For example, mutant p62 shows a lower affinity for LC3, reducing the efficiency of autophagy [17]. Moreover, mutations in Tank Binding Kinase 1 may result in impaired autophagy (and thus mitophagy), leading to the accumulation of protein and mitochondria in ALS [18]. Moreover, lysosomal dysfunction was found in ALS, while lysosomal deficits accompanied by impaired autophagic degradation happen progressively in a mutant SOD1 transgenic mouse model of ALS [19]. Finally, VCP seems to affect several pathways, such as autophagosome maturation and mitophagy [20]. 

These recent findings support the hypothesis of the involvement of mitochondria in the pathogenesis of ALS, but their degradation mechanisms are still unknown. Thus, this work aimed to investigate mitochondrial morphology, dynamism, and mitophagy in patients’ peripheral blood mononuclear cells (PBMCs), which may provide useful information on physiopathology in sporadic patients [21,22,23,24]. We provided evidence of mitophagy impairment in ALS; thus, we dissected the pathway finding a dysregulation of lysosomes. Finally, we proposed potential therapeutic strategies that could be effective for ALS treatment.

## 2. Materials and Methods

### 2.1. Patients’ Enrolment

Patients (*N* = 32; male: 17, female 15, mean age: 67.8 ± 9.9) affected by ALS were enrolled at the “IRCCS Mondino Foundation” in Pavia (Italy). Experiments were done using PBMCs isolated from sALS patients. ALS diagnosis was made according to the revised El Escorial Criteria [25]. SALS individuals harboring mutations in the SOD1, FUS/TLS, TARDBP, C9ORF72, VCP, and OPTN genes were excluded from this study. Sex- and age-matched healthy volunteers (*N* = 32; male: 18, female 14, mean age: 69.9 ± 6.4), free from any pharmacological treatment, were recruited at the Transfusion Centre of the IRCCS Policlinico S. Matteo Foundation in Pavia (Italy). Healthy volunteers were all unrelated and the healthy phenotype was confirmed by interviews based on personal health history. The study design was examined by the ethical committee of the enrolling Institutions (p-20180034329).

### 2.2. Isolation of PBMCs from ALS Patients and Healthy Controls

PBMCs were immediately isolated from peripheral venous blood by Histopaque^®^-1077 (Sigma-Aldrich, St. Louis, MO, USA) following the manufacturer’s instructions. Cells viability was assessed by trypan blue exclusion test using the automated T20 Cell Counter (Bio-Rad Laboratories, Hercules, CA, USA). Aliquots of PBMCs were collected from each subject and processed for the following experiments.

### 2.3. Sample Preparation for Transmission Electron Microscopy (TEM) 

Approximately 3 × 10^6^ cells were washed in phosphate-buffered saline (1X PBS) and incubated with the fixing solution (2,5% glutaraldehyde and 2% paraformaldehyde in cacodylate buffer, pH 7.3) for 4 h at 4 °C, followed by a post-fixation in 1.5% osmium tetroxide for 1 h at room temperature (RT) and Epon-Araldite embedding [26]. Ultrathin sections (~70 nm thick) were cut from the resin blocks and stained with uranyl acetate/lead citrate and observations were performed using conventional electron microscopy at the University of Pavia facility (Italy). The area of mitochondria was measured using the free ImageJ v.1.53g (W. Rasband, NIH, Bethesda, MD, USA) software (https://imagej.nih.gov/ij/, accessed on 1 February 2021).

### 2.4. Extraction of Mitochondrial Fraction

According to manufacturer instructions, the mitochondrial fraction was isolated using the Cytochrome C Releasing Apoptosis Assay Kit (Abcam, Cambridge, UK). Briefly, 6 × 106 cells were centrifuged at 600× *g* at 4 °C and pellets were washed in ice-cold 1X PBS and centrifuged again. The supernatant was discarded and cells were resuspended in 1X Cytosol Extraction Buffer. Samples were incubated on ice and then centrifuged at 10,000× *g* at 4 °C. The supernatant was collected as the cytosolic fraction. The remaining pellet was resuspended in Mitochondrial Extraction Buffer, plus 1 mM DTT and 1% PIC, and stored at −80 °C as a mitochondrial fraction. The protein content of each extracted fraction was then quantified by BCA assay (Sigma-Aldrich, St. Louis, MO, USA) and measured with Nanodrop 2000 (Thermo Fisher Scientific, Waltham, MA, USA). 

### 2.5. Western Blotting Analysis

Western blotting analysis was performed by SDS–polyacrylamide gel electrophoresis (SDS-PAGE). 50 μg of proteins were loaded into 10% SDS–PAGE gel. After electrophoresis, samples were transferred to PVDF membrane (Trans-blot, Bio-Rad Laboratories, Hercules, CA, USA) using a liquid transfer apparatus. Membranes were treated with a blocking solution (5% of non-fat dry milk in TBS-T buffer, 10 mM Tris-HCl, 100 mM NaCl, 0.1% Tween, pH 7.5) to block unspecific protein binding sites and incubated with primary antibody overnight (ON) at 4 °C (Table 1).

Immunoreactivity was detected using donkey anti-rabbit or anti-mouse secondary peroxidase-conjugated antibody (dilution 1:5000; Little Chalfont, UK) and bands were visualized using an enhanced chemiluminescence detection kit (ECL Select, Little Chalfont, UK). Both primary and secondary antibodies were removed from the membrane using stripping solution (100mM glycine, 0.1% NP40, 1% SDS, pH 2.2) and then processed as described above. Densitometric analysis of the bands was performed using the free ImageJ software (http://rsb.info.nih.gov/ij/, accessed on 1 February 2021).

### 2.6. Organelles Analysis

About 1 × 10^5^ cells were placed on a poly-L-lysine slide (Thermo Fisher Scientific, Waltham, MA, USA) and incubated for 30 min to allow their attachment to the slide. For mitochondria staining cells were incubated for 30 min at 37 °C with 100nM of MitoTracker™ Red CMXRos (Thermo Fisher Scientific, USA), for lysosomes staining cells were incubated for 30 min at 37 °C with 50 nM of LysoTracker™ (Thermo Fisher Scientific, USA) and for endoplasmatic reticulum (ER) staining cells were incubated for 30 min at 37 °C with 500nM of ER-Tracker™ (Thermo Fisher Scientific, USA). Cells were washed 3 times with 1X PBS and fixed with paraformaldehyde (PFA) 4%/1× PBS for 15 min. Cells were rinsed with 1X PBS for 3 times. Slides were mounted with Prolong^®^ Gold antifade reagent with DAPI (Thermo Fisher Scientific, USA). Confocal imaging acquired images using an Axioimager 2 (Zeiss, Oberkochen, Germany) equipped with a ×63 oil-immersion lens.

### 2.7. Cytofluorimetry Analysis

1 × 10^5^ living PBMCs were resuspended in a staining mix composed of 1X PBS plus 0.2% of BSA added with 200 nM of MitoTracker™ Green (Thermo Fisher Scientific, USA) and 200 nM of TMRE (Abcam, UK). Successively, cells were incubated for 20 min at 37 °C, followed by centrifugation at 600× *g*. After centrifugation, the supernatant was discarded, and the pellet was resuspended in 300 μL of 1× PBS. Finally, cells suspension was analyzed by flow cytometer BD FACSCanto II (BD Biosciences, Franklin Lakes, NJ, USA).

### 2.8. Immunofluorescence Cells Analysis

1 × 10^5^ cells were placed on a poly-L-Lysine slide (Thermo Fisher Scientific, USA) and incubated at 37 °C to allow cell attachment to the slide. Cells were rinsed with 1X PBS and then fixed using a solution of 4% PFA/1X PBS [27]. Fixed cells were washed with 1X PBS and treated with a blocking solution (5% normal goat serum in 0.1% Tween-PBS) for 1 h to block unspecific protein binding sites; cells were then incubated o/n at 4 °C with primary antibodies: rabbit polyclonal anti-LC3 (1:250 dilution; Sigma-Aldrich), mouse monoclonal anti-PINK1 (1:250 dilution; Abcam, UK). Cells were washed with 1X PBS and incubated at RT for 1 h with secondary antibodies: CFTM 594 goat anti-mouse (1:700 dilution, Sigma-Aldrich, Italy) and CFTM 488A goat anti-rabbit (1:700 dilution; Sigma-Aldrich, Italy). Both primary and secondary antibodies were prepared in the blocking buffer. Finally, samples were washed with 1X PBS, mounted with Prolong^®^ Gold antifade reagent with DAPI (Thermo Fisher Scientific, USA), dried, nail-polished, and analyzed by confocal microscopy (Leica Microsystems Srl, Wetzlar, Germany). 

### 2.9. Cells Treatment

1 × 10^6^ PBMCs were plated into a 6-well plate and cultured in RPMI medium added with 20% Fetal Bovine Serum (FBS), 2 mM L-glutamine, 100 U/mL penicillin, 10 mg/mL streptomycin in an atmosphere of 5% CO_2_ and 95% humidity. Cells were treated for 3 h in medium added with 10 nM of Rapamycin (Sigma-Aldrich), 24h in medium added with 2.5 mM NH4Cl, and 48h in medium added with 100 mM trehalose (Sigma-Aldrich).

### 2.10. Power and Statistical Analysis

The sample size was calculated by performing a power analysis with the following conditions: μ1 = 1, μ2 = 1.5, σ = 0.7, α = 0.5, power = 0.80. The conditions were chosen considering our preliminary results on smaller populations. We used *N* = 32 for western blot analysis, while for microscopies experiments, we used *N* = 3 (5 fields for each sample) for each group.

All the experiments were performed at least three times. Values were expressed as means ± S.D. Statistical analysis was performed by Student’s t-test and One-Way Analysis of Variance (ANOVA) followed by Newman-Keuls Multiple Comparison as a post-hoc test (GraphPad Prism version 5, GraphPad Software, San Diego, CA, USA). Values were considered statistically significant when *p* values were <0.05.

## 3. Results

### 3.1. Mitochondria Alteration in PBMCs of sALS Patients

**Figure 1:**
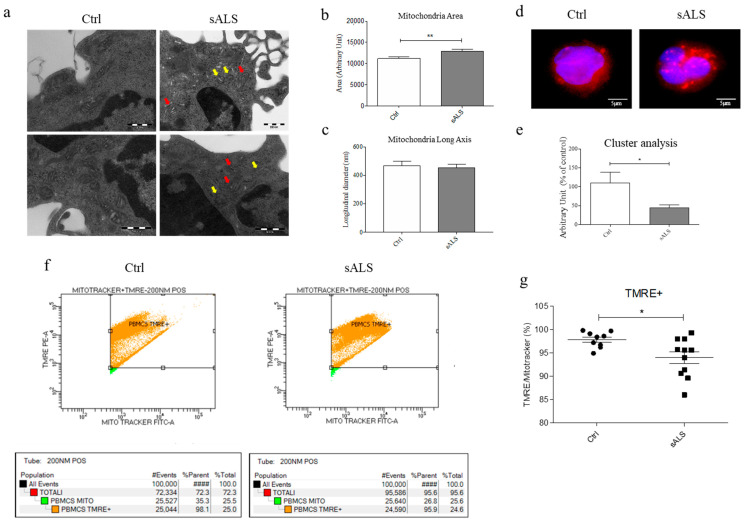
PBMCs of sALS patients show alteration in mitochondrial structural morphology. (**a**) TEM analysis of mitochondrial structural morphology: yellow arrows indicate mitochondria with deformed cristae, while red arrows indicate degenerated mitochondria; scale bar: 250 nm. *N* = 3 (**b**) Measure of the mean area of mitochondria (ImageJ software), in sALS patients’ mitochondria are larger with respect to controls (** *p* < 0.01). *N* = 3 (**c**) Measure of the long mitochondrial axis (nm). *N* = 3 (**d**) Analysis of mitochondrial spatial organization by immunofluorescence using MitoTracker™ Red Blue: DAPI, Red: MitoTracker™. Scale bar: 5 µm. *N* = 3 (**e**) Analysis of clusterization. Data are reported as mean (* *p* < 0.05) of the percentage of number/size in sALS PBMCs respect to controls. *N* = 3 (**f**,**g**) Flow Cytometry analysis of the ratio between healthy and damaged mitochondria. Data are reported as the mean (* *p* < 0.05) of the percentage of healthy mitochondria in sALS PBMCs with respect to controls. MitoTracker™ marks all mitochondria, while TMRE marks the healthy ones. *N* = 12.

To understand the involvement of mitochondria in ALS pathology using sALS derived PBMCs, we first investigated using TEM if, in these cells, the mitochondrial structural morphology is altered. Several differences in mitochondrial shape and internal structure are clearly appreciable in samples of healthy control subjects and sALS patients. In controls PBMCs, mitochondria have a long axis of about 500 nm, normal cristae, and an absence of vacuoles in the matrix. On the contrary, sALS mitochondria are characterized by relevant cristae deformation (Figure 1a). In sALS PBMCs the mean area of mitochondria (Figure 1b) resulted significantly larger than controls PBMCs (** *p* < 0.01). No differences were found in long axis measurement between controls and sALS PBMCs (Figure 1c). Because of the poor quality, further analyses were performed. Alterations in the mitochondrial spatial organization within the cytoplasm have been investigated by immunofluorescence (IF) using MitoTracker™ Red CMXRos (Thermo Fisher Scientific, USA). While in control PBMCs, mitochondria are homogeneously distributed in the whole cytoplasmic compartment, in PBMCs of sALS patients, mitochondria appear to be clusterized (Figure 1d). We measured both the size and number of fluorescent stains and then reported the ratio number/size. Indeed, we expect a reduced number and an increased size of fluorescent stain (thus, reduced ratio) in case of clusterization. We have a statistically significant decrease in the number/size ratio in sALS PBMCs compared to Ctrl (Figure 1e). These data suggest that in PBMCs of sALS morphologically altered and damaged mitochondria accumulate in large structures, suggesting an altered mitochondria turnover and clearance. To understand whether mitochondria are still functional, we evaluated the ratio between damaged and healthy mitochondria by flow cytometry. We stained fresh PBMCs with MitoTracker™ Green FM, which enters both healthy and damaged mitochondria, and with TMRE, which marks only the healthy ones because it does not enter when mitochondria are damaged or depolarized. While the total number of mitochondria did not change between sALS and healthy control PBMCs (MitoTracker™ ), a statistically significant decrease was found in the TMRE/MitoTracker™ ratio (* *p* < 0.05) in PBMCs of sALS patients compared to the healthy controls (Figure 1f,g). The data suggest an accumulation of damaged/depolarized mitochondria in sALS samples. Thus, to further investigate the process of fusion and fission, and apoptosis, we analyzed the expression levels of DRP1, OPA1, MFN1, Cyt C, and BCL-2 [28,29] proteins by WB both in the cytoplasmic and mitochondrial compartment of sALS and healthy control PBMCs. We did not find any statistically significant difference in the expression of these markers between the two groups (Figure S1a–e), suggesting that in PBMCs of sALS patients, there is no dysregulation in mitochondrial fission and fusion or mitochondria-mediated apoptosis. Interestingly, we found a decreasing trend of the ratio long-OPA1/short-OPA1 in sALS compared to Ctrl, suggesting activation of mitophagy in sALS PBMCs (Figure S1f) [30].

### 3.2. Upregulation of the Mitophagy Pathway in PBMCs of sALS Patients

**Figure 2:**
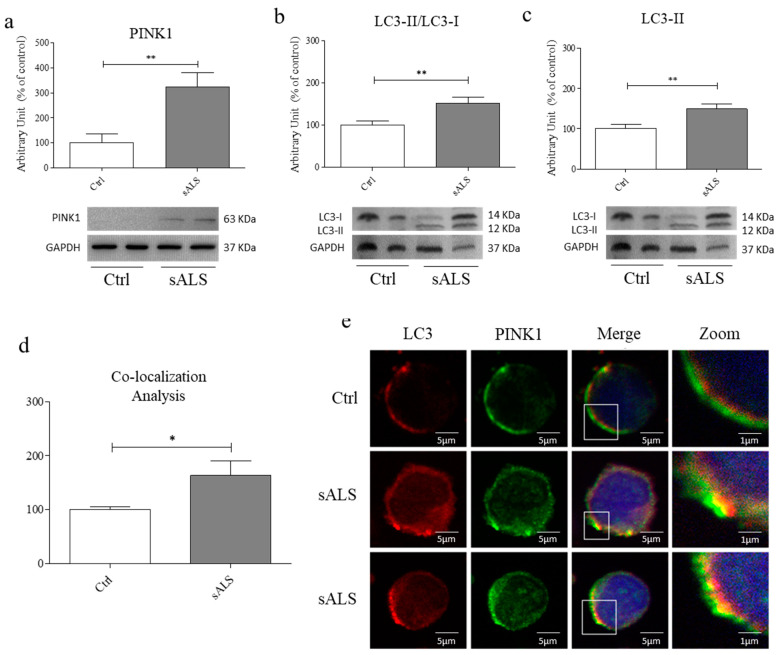
Mitophagy pathway increases in PBMCs of sALS patients. (**a**) In PBMCs of sALS patients, we observed a statistically significant increase in PINK1 expression levels (** *p* < 0.01). *N* = 32 (**b**) In PBMCs of sALS patients we observed a statistically significant increase in LC3-II/LC3-I ratio (** *p* < 0.01). *N* = 32 (**c**) In PBMCs of sALS patients we observed a statistically significant increase in LC3-II expression levels (** *p* < 0.01). We used GAPDH as a loading control. *N* = 32 (**d**,**e**) Analysis of the co-localization (merge) between LC3 and PINK1 in PBMCs of healthy controls and sALS patients by confocal microscopy analysis. We found an increased co-localization in sALS PBMCs (* *p* < 0.05). Red: LC3, Green: PINK1. Magnification: 63X, scale bar: 5 µm. Zoom: X4, scale bar 1 µm. *N* = 3.

As stated before, mitophagy is a form of macroautophagy in which damaged mitochondria are sequestered into double-membrane autophagosomes going to fuse with the lysosome. This is a critical quality control mechanism allowing the maintenance of the correct mitochondrial homeostasis (functionality and network) [31,32]. Thus, we analyzed by WB the level of PINK1 and found a statistically significant increase in sALS PBMCs with respect to Ctrl (** *p* < 0.01) (Figure 2a). Then, we observed in sALS PBMCs a statistically significant increase in LC3-II/LC3-I ratio compared to Ctrl (** *p* < 0.01) (Figure 2b). We also evaluated the expression of LC3-II and we found a significant increase (** *p* < 0.01) (Figure 2c) in sALS PBMCs compared to Ctrl. This evidence suggests an upregulation in mitophagy in sALS patients PBMCs.

These observations suggest that in sALS PBMCs, the mitophagy pathway is impaired. To corroborate this hypothesis, we analyzed the co-localization between LC3 and PINK1 in PBMCs of Ctrl and sALS by confocal microscopy analysis. We found an increased co-localization between LC3 and PINK1 in sALS PBMCs compared to Ctrl (Figure 2d–e), proving that in sALS patients, mitophagy is impaired. Moreover, in this case, the quality of the images did not show any other information.

### 3.3. Inhibition of Fusion between Autophagosomes and Lysosomes

**Figure 3:**
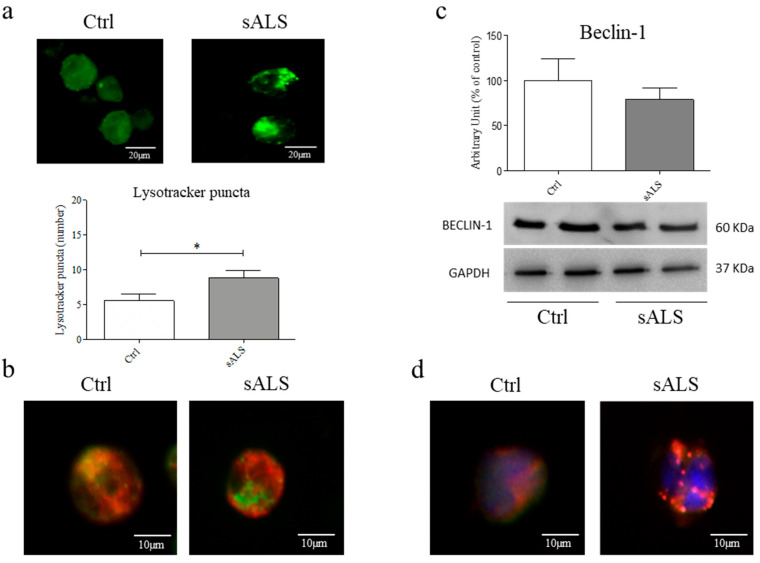
Inhibition of fusion between autophagosomes and lysosomes. (**a**) Immunofluorescence analysis with LysoTracker™ marker revealed an accumulation of lysosomes in sALS patients’ PBMCs. We found a statistical increase in the number of puncta in sALS compared to control (* *p* < 0.05). Scale bar: 20 µm. *N* = 3 (**b**) Immunofluorescence analysis with LC3 and LysoTracker™ revealed that the fusion between lysosomes and autophagosomes is inhibited. Green: LysoTracker™; Red: LC3. Scale bar: 10 µm. *N* = 3 (**c**) WB analysis revealed no statistically significant differences between levels of Beclin-1 in sALS patients and control. GAPDH was used as a loading control. *N* = 32 (**d**) Immunofluorescence analysis with ER-Tracker™ revealed that mitochondria (Red) and ER (green) do not co-localize. Scale bar: 10 µm. *N* = 3.

Since damaged mitochondria are degraded by mitophagy, we tested the efficiency of lysosomes. We evaluated lysosome distribution and number by quantifying green puncta in Lysotraker stained PBMCs. We found lysosomes accumulation in the cytoplasm of sALS patient PBMCs, while a homogenous distribution of lysosomes was found in PBMCs of healthy controls (Figure 3a). Moreover, to test the efficiency of mitophagy, we investigated the fusion between autophagosomes and lysosomes by detecting the co-localization between LC3 and LysoTracker™. We did not find any co-localization between LC3 and LysoTracker™, indicating that the fusion process could inhibit sALS patients’ PBMCs (Figure 3b). We also wondered if the mitophagy was altered at the beginning of the mitophagy cascade; thus, we analyzed using WB the expression levels of Beclin-1, a protein involved in mitophagy trigger [33]. No statistically significant differences were found between healthy controls and sALS patients (Figure 3c). Moreover, we did not observe any alteration of the endoplasmic reticulum (ER) (Figure 3d), concluding that only the fusion between autophagosomes and lysosomes is impaired.

### 3.4. Rapamycin and NH4Cl Confirmed the Inhibition of Autolysosomes Generation

**Figure 4:**
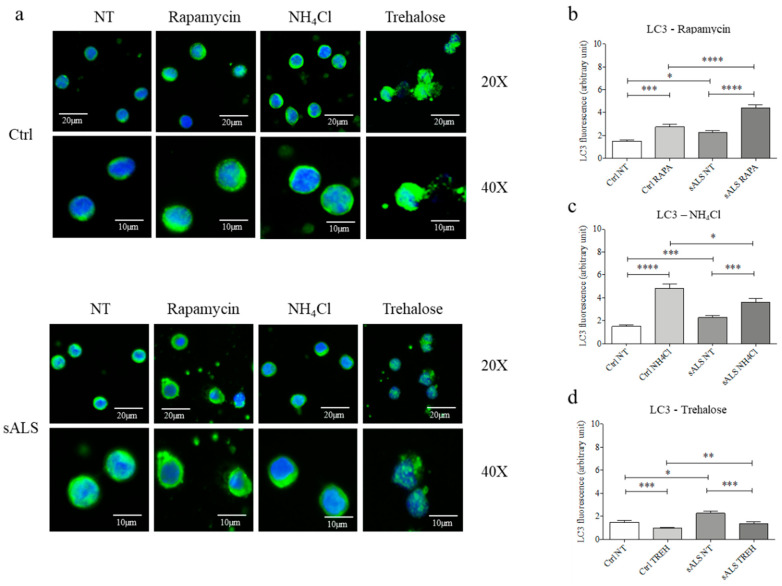
Dissection of autolysosomes generation sALS PBMCs vs. Controls. (**a**) Immunofluorescence analysis with LC3 revealed an accumulation of autophagosomes in sALS patients’ PBMCs in NT conditions. After rapamycin treatment we found an increased accumulation of LC3 in sALS PBMCs compared to controls. On the other hand, we found a higher increase in LC3 after NH4Cl treatment in healthy controls respect to sALS patients. Scale bar 20X: 20 µm, Scale bar 40X: 10 µm. *N* = 3 (**b**) ImageJ analysis of LC3 after rapamycin treatment. We found statistically significant differences between Ctrl NT and sALS NT (* *p* < 0.05), Ctrl NT and Ctrl RAPA, sALS NT and sALS RAPA (*** *p* < 0.001), and Ctrl RAPA and sALS RAPA (**** *p* < 0.0001). (**c**) ImageJ analysis of LC3 after NH4Cl treatment. We found statistically significant differences between Ctrl NT and sALS NT, Ctrl NH4Cl and sALS NH4Cl (* *p* < 0.05), Ctrl NT and Ctrl NH4Cl (**** *p* < 0.0001), and sALS NT and sALS NH4Cl (*** *p* < 0.001). (**d**) ImageJ analysis of LC3 after trehalose treatment. We found statistically significant differences between Ctrl NT and sALS NT (* *p* < 0.05), Ctrl TREH and sALS TREH (** *p* < 0.01), Ctrl NT and Ctrl TREH, and sALS NT and sALS TREH (*** *p* < 0.001).

To further confirm the inhibition of the fusion between autophagosomes and lysosomes, we treated cells with rapamycin (10 nM), which induces autophagy by inhibiting mTOR [34]. Using IF, we observed that 3 h of rapamycin treatment significantly increased LC3 level in sALS PBMCs (* *p* < 0.05) compared to healthy control PBMCs (Figure 4a,b and Figure S2), suggesting that mitophagy activation is higher in sALS than controls. On the contrary, NH4Cl (2.5mM) inhibits the degradation of LC3 by autophagolysosome acidification inhibition [35]. After 24 h of treatment, we found a lower increase in LC3 level in sALS PBMCs (* *p* < 0.05) compared to healthy control PBMCs (Figure 4a,c and Figure S2). Finally, we treated cells with trehalose (100mM) for 48 h, as this compound induces rapid and transient lysosomal damage [28]. We found a higher decrease in LC3 level in sALS PBMCs (* *p* < 0.05) compared to healthy control PBMCs (Figure 4a,d and Figure S2). All these data led us to suppose that in sALS PBMCs, the partial inhibition of both the fusion between autophagosomes and lysosomes and the formation of autolysosomes causes the accumulation of autophagosomes. 

### 3.5. Trehalose Treatment Induces the Lysosomes Clearance

**Figure 5:**
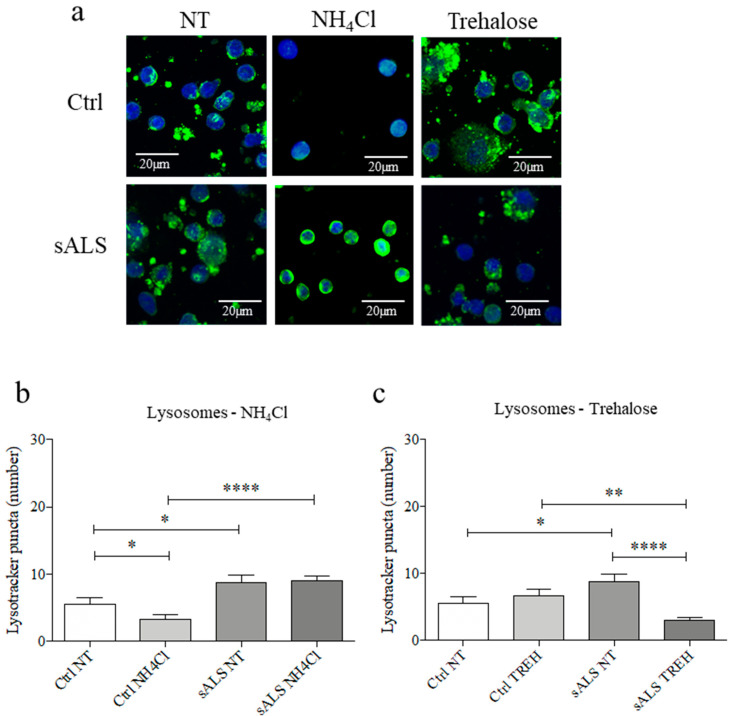
Trehalose proves to help to remove accumulated lysosomes. (**a**) Immunofluorescence analysis of LysoTracker™ in sALS patients and healthy controls treated and non-treated with NH4Cl or Tehalose; Scale bar: 20 µm. *N* = 3 (**b**) ImageJ quantification of LysoTracker™ puncta. We found statistically significant differences between Ctrl NT and sALS NT, CTRL NT and CTRL NH4Cl (* *p* < 0.05) and Ctrl NH4Cl and sALS NH4Cl (**** *p* < 0.0001). (**c**) ImageJ analysis of LysoTracker™ puncta. We found statistically significant differences between Ctrl NT and sALS NT (* *p* < 0.05), CTRL TREH and SALS TREH (** *p* < 0.01) SALS NT and sALS TREH (**** *p* < 0.0001).

To confirm the engulfment of autophagolysosome formation, we measured lysosomes puncta by labeling with LysoTracker™ in both NH4Cl and trehalose cells. After NH4Cl treatment, we found a decrease in lysosomes number (* *p* < 0.05) in healthy control cells, while we did not find any differences in sALS PBMCs (Figure 5a,b). Intriguingly, we found a strong decrease (**** *p* < 0.0001) of lysosomes number in PBMCs of sALS patients after trehalose treatment (Figure 5a,c). These data suggest that in a pathological condition, such as ALS, trehalose could help to remove accumulated lysosomes.

## 4. Discussion

In neurodegenerative diseases, such as ALS, mitochondrial dysfunctions play important roles in triggering disease as mitochondria are needed for physiological cell metabolism and survival. In this study, we investigated the mitophagy impairment in our peripheral model of ALS, represented by patients’ PBMCs, which were proved to be a good model to study the progression of neurodegeneration in patients [23,36]. 

Firstly, in PBMCs of sALS patients, we found an alteration in mitochondrial morphology by TEM analysis. As PBMCs are very small, in the future, it would be interesting to evaluate the same alteration in other cellular models, with the possibility to have a higher resolution of TEM images. The alteration of mitochondrial morphology, e.g., fragmented network, swelling, and augmented cristae, were found in both animal [37] and cellular models of the disease, such as skeletal muscle [9], spinal motor neurons, lymphoblastoid cell line [36]. Our data confirm the involvement of mitochondria as the hallmark of ALS in PMBCs of affected patients. Since mitochondria have a bigger area in sALS patients compared to controls, we decided to study mitochondrial dynamism. We expected an increase in fusion proteins and a decrease in fission ones, but curiously, we did not find any statistically significant alteration in both fusion and fission pathways. The absence of mitochondrial dynamism impairment suggested that we investigate another important pathway in which mitochondria are involved, notably apoptosis. In particular, considering that both intrinsic and extrinsic apoptotic cascades stimulate the release of Cyt C from the mitochondrial membrane to the cytoplasm, we evaluated the release of Cyt C in the cytoplasmic compartment in PBMCs. In sALS patients, we did not find any changes compared to healthy controls suggesting that apoptosis is not implicated in the mitochondria impairment we found.

Remarkably, we found accumulation and clusterization of mitochondria in the cytoplasm of sALS PBMCs by MitoTracker™ analysis. To confirm this data, we analyzed the ratio between healthy and damaged mitochondria by flow cytometry, and we found a statistically significant decrease in TMRE/MitoTracker™ ratio in sALS PBMCs with respect to controls and that the percentage of TMRE+ mitochondria is reduced in sALS PBMCs. These findings suggest that in sALS PBMCs damaged or depolarized mitochondria are not completely removed, causing impairment in cellular metabolism. Thus, we analyzed the last critical pathway involving mitochondria: their degradation and removal by mitophagy. Impairment of the mitophagy system has been reported in sALS patients, with many vesicles in the cytoplasm of normal motor neurons, which became more evident in degenerated motor neurons [11]. The involvement of mitophagy in ALS is supported by the study of ALS-related genes, e.g., ALS2/alsin, which seem to contribute to the trafficking of endosome-autophagosome [38]. Mitophagy was found to be impaired, as suggested by several studies. It seems that mutant SOD1 impairs mitochondrial retrograde axonal transport, resulting in a delay in the mitophagy flux [39]. Moreover, mitophagy induction in ALS is supported by the increase in the LC3-II/LC3-I ratio in neurons of SOD1 transgenic mice [40]. While an accumulation of autophagosomes that contain mitochondria was found in human spinal cord ALS samples [41]. Thus, we investigated two markers of both autophagy e mitophagy by WB, observing an increased LC3-II/LC3-I ratio and PINK1 expression. LC3-II/LC3-I ratio is the most used macroautophagy marker because the conversion of LC3-I to its lipidated LC3-II form is essential for the nucleation of the autophagosomes [42]. Moreover, during Parkin-dependent mitophagy, damaged mitochondria are removed by the interaction between PINK1 and Parkin. PINK1 and Parkin flag mitochondria to be eliminated, accumulating in the outer mitochondrial membrane (OMM) and allowing their clearance [43,44]. Moreover, the co-localization between LC3 and PINK1 suggests this alteration in the mitophagy pathway, but further investigation is required because of the quality of confocal images. As the mitophagy pathway is divided into 4 subsequent steps, nucleation/elongation of autophagosomes, their maturation, and fusion with lysosomes and degradation, we wondered if the impairment of mitophagy pathway occurs in the fusion of mitochondria containing autophagosomes and lysosomes. Firstly, we analyzed the presence and the efficiency of lysosomes by immunofluorescence analysis that reveals an accumulation of lysosomes in sALS patient PBMCs. This result led us to suppose that the fusion between autophagosomes and lysosomes is inhibited, so we proceeded with the analysis of these mechanisms by immunofluorescence analysis using LC3 and LysoTracker™. No co-localization of LC3 and LysoTracker™ was found in sALS PBMCs, suggesting the inhibition between autophagosomes and lysosomes. Then, we measured the levels of Beclin-1, an important regulator of early autophagosome formation, and the possible co-localization between ER and mitochondria using ER-Tracker™ and MitoTracker™ markers. Beclin-1 plays an important role in early autophagosome formation. As Belcin-1 remained unchanged, the initial phases of mitophagy are not impaired. To further confirm our hypothesis, we treated cells with two drugs: rapamycin, which induces autophagy by inhibiting the mTOR pathway [45] and NH4Cl, which inhibits the degradation of LC3 usually caused by the acidification of autolysosomes [46]. The inhibition of the fusion between autophagosomes and lysosomes, which occurs in ALS, causes a higher increase in the accumulation of LC3 in rapamycin-treated cells because of the reduced capacity to degrade LC3. On the contrary, we demonstrated that the inhibition of the degradation of autophago-lysosomes due to NH4Cl causes a lower increase in LC3 because ALS inhibits the previous step of the autophagic pathway. Moreover, we found that NH4Cl did not change the amount of lysosomes in ALS, while they tended to decrease in healthy controls.

The balance between synthesis and degradation of mitochondria is fundamental to maintaining cellular homeostasis, and the modulation of mitophagy represents a promising way of intervention. Trehalose, also known as mycose, is a natural disaccharide that modulates autophagy [47], causing rapid and transient lysosomal damage [48]. We found that trehalose treatment decreases LC3 levels, probably inducing the degradation of accumulated autophagolysosomes. The decrease seems higher in sALS than in healthy control because autophagolysosomes accumulate more. Then, we treated with trehalose PBMCs of patients and healthy controls to confirm the inhibition of autophago-lysosome formation. In non-treated sALS PBMCs, we have observed a higher number of lysosomes, confirming their accumulation. Interestingly, after trehalose treatment, we found a significant decrease in the number of lysosomes in PBMCs of sALS patients and an increase in healthy control PBMCs. After trehalose treatment, the reduction of the accumulated lysosome and the level of marker proteins in sALS PBMCs suggested that trehalose treatment could induce recovery of the mitophagy pathway. Moreover, our finding suggests a higher susceptibility of ALS lysosomes compared to healthy control.

## 5. Conclusions

Considering these findings, we conclude that the accumulation of damaged mitochondria in PBMS of sALS patients is due to an impairment of autophagosome trafficking that is partially reverted with trehalose. The presence of altered and dysfunctional mitochondria may contribute to the pathogenesis of ALS, unraveling a possible new therapeutic target to delay the disease. In particular, the use of some drugs, such as trehalose, can be a starting point for new treatments.

## Figures and Tables

**Table 1:** Antibodies used in the WB analysis. We reported the host species, the dilution, and the brand of each antibody.

Antibodies	Species	Dilution	Brand
LC3 (SAB1305552)	mouse	1:500	Sigma-Aldrich
PINK1 (ab23707)	rabbit	1:1000	Abcam
Beclin-1 (ab62557)	rabbit	1:1000	Abcam
DRP1 (ab56788)	mouse	1:500	Abcam
MFN1 (ab57602)	mouse	1:1000	Abcam
OPA1 (ab157457)	rabbit	1:1000	Abcam
CYT C (ab65311)	mouse	1:200	Abcam
BCL-2 (ab59348)	rabbit	1:200	Abcam

## Data Availability

The datasets generated and analyzed during the current study are available after a reasonable request to the corresponding author in the Zenodo repository, DOI: 10.5281/zenodo.5541390.

## Supplementary Materials

The following supporting information can be downloaded at: https://www.mdpi.com/article/10.3390/cells11081272/s1, Figure S1: WB analysis of involved in mitochondrial fusion (**a**,**b**), fission (**c**), and apoptosis (**d**,**e**); the ratio of long OPA1 and short OPA1. *N* = 32 Figure S2: The ratio of LC3 values between untreated and treated PBMCs in both Ctrl and sALS was evaluated. *N* = 3
