# Supplementary material for: Lysosomes Dysfunction Causes Mitophagy Impairment in PBMCs of Sporadic ALS Patients
Source: Cells. 2022 Apr 9;11(8):1272. doi: 10.3390/cells11081272 (PMC9030813; doi:10.3390/cells11081272)
Supplement: Supplementary file 1 [file cells-11-01272-s001.zip › cells-1641233-supplementary.pdf]

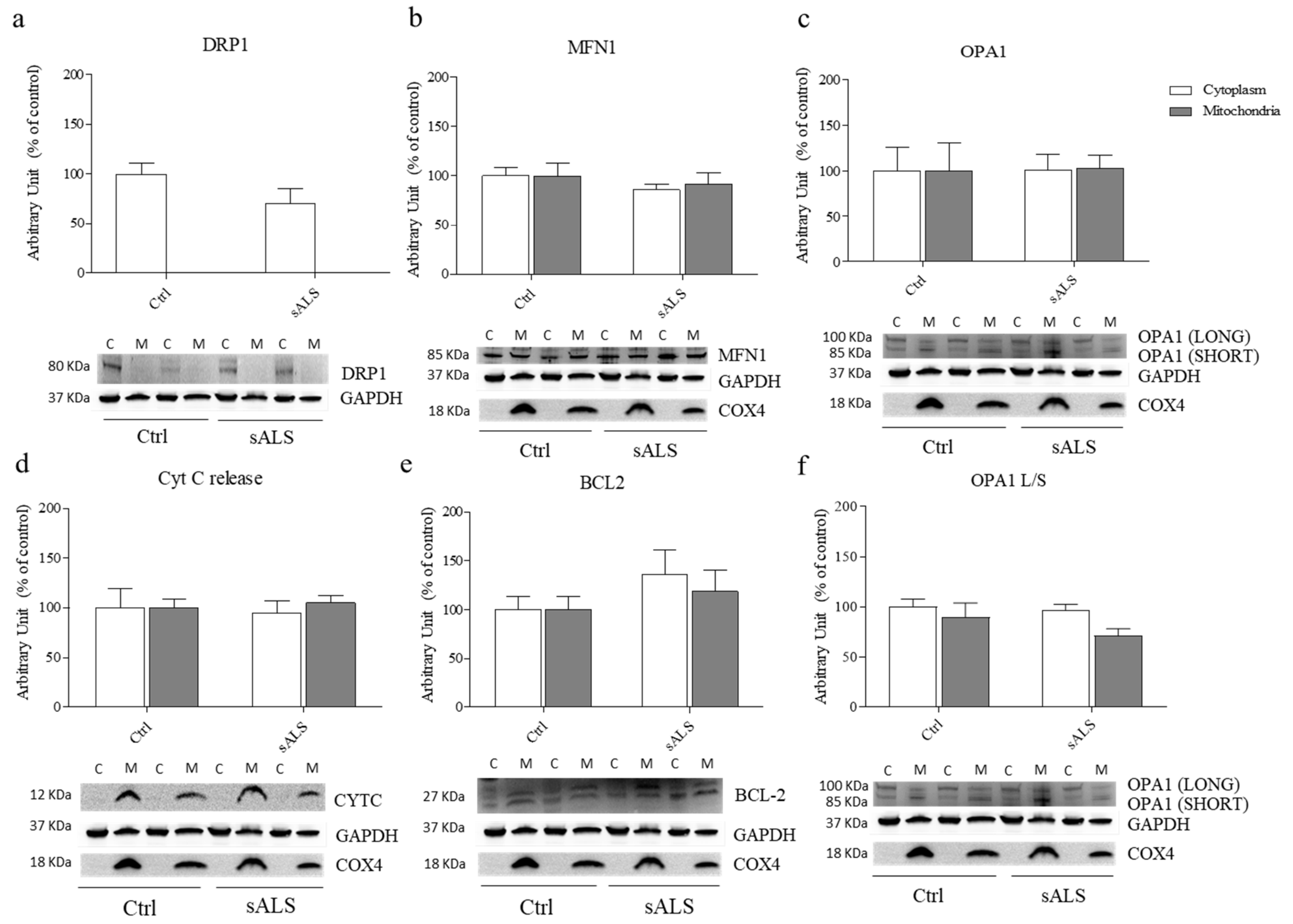

Figure S1. WB analysis of involved in mitochondrial fusion (a,b), fission (c), and apoptosis (d,e); the ratio of long OPA1 and short OPA1. N = 32

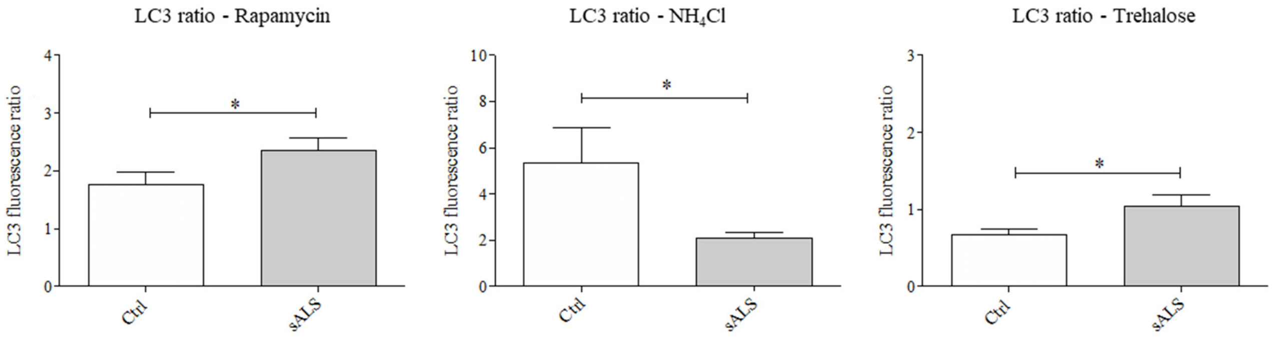

Figure S2. The ratio of LC3 values between untreated and treated PBMCs in both Ctrl and sALS was evaluated. *N* = 3
